# Interstitial lung disease induced by apalutamide therapy for castration‐resistant prostate cancer: A report of a rare case

**DOI:** 10.1002/iju5.12420

**Published:** 2022-03-03

**Authors:** Fumiaki Kirishima, Yoshinori Shigematsu, Kanao Kobayashi

**Affiliations:** ^1^ Department of Urology Chugoku Rosai Hospital Kure City Hiroshima Japan

**Keywords:** apalutamide, dyspnea, interstitial lung disease, methylprednisolone, prostate cancer

## Abstract

**Introduction:**

Apalutamide is a new second‐generation anti‐androgen agent approved in 2019 for the treatment of metastatic, castration‐sensitive, and non‐metastatic, castration‐resistant prostate cancer. We herein report a case of apalutamide‐induced interstitial lung disease.

**Case presentation:**

A 74‐year‐old Japanese male patient with non‐metastatic, castration‐resistant prostate cancer commenced hormonal therapy with apalutamide (240 mg/day orally) after 46 months of maximal androgen blockade therapy with bicalutamide and leuprorelin. Thirty‐five days following therapy initiation with apalutamide, he was hospitalized because of dyspnea. Chest computed tomography showed diffuse bilateral interstitial infiltrates and ground‐glass opacities in the upper and lower lobes of the lungs. Following a diagnosis of drug‐induced interstitial lung disease resulting from apalutamide treatment, the treatment with apalutamide was stopped. Steroid therapy was initiated, and the dyspnea resolved.

**Conclusion:**

Clinicians should be aware that apalutamide, and other drugs in general, can cause drug‐induced interstitial lung disease within 3 months.

**Abbreviations**
AR: androgen receptor
CT: computed tomography
DLST: drug‐induced lymphocyte stimulation test
ILD: interstitial lung disease
KL‐6: Krebs von den Lungen‐6
PSA: prostate‐specific antigen

**Keynote message**

The incidence of interstitial lung disease (ILD) associated with second‐generation antiandrogens is extremely rare. Apalutamide is new to the market; thus, there are few reports of side effects. Since ILD can be a serious condition, urologists should be aware of the risk of drug‐induced ILD with apalutamide.

## Introduction

Androgen‐signaling–targeted inhibitors, such as enzalutamide, abiraterone, and apalutamide, are widely used in the treatment of prostate cancer. However, only a limited number of cases of drug‐induced ILD have been reported as a result of AR inhibitors, particularly involving bicalutamide,^1^, ^2^, ^3^, ^4^, ^5^ abiraterone,^6^ and apalutamide.^7^ Herein, we report a case of drug‐induced ILD that occurred following the treatment with apalutamide, a recently approved AR inhibitor.

## Case report

A 69‐year‐old Japanese man with cT3N0M0 prostate cancer started maximal androgen blockade therapy with bicalutamide and leuprorelin in June 2015. He did not want local therapy such as surgery and radiation therapy. His initial PSA level and Gleason score were 61.05 ng/mL and 4 + 5=9, respectively. The patient had no metastasis, and his PSA level was as low as 1.657 ng/mL in April 2020; however, his PSA doubling time was less than 6 months. Thus, at 74 years of age, treatment with apalutamide was initiated.

**Fig. 1:**
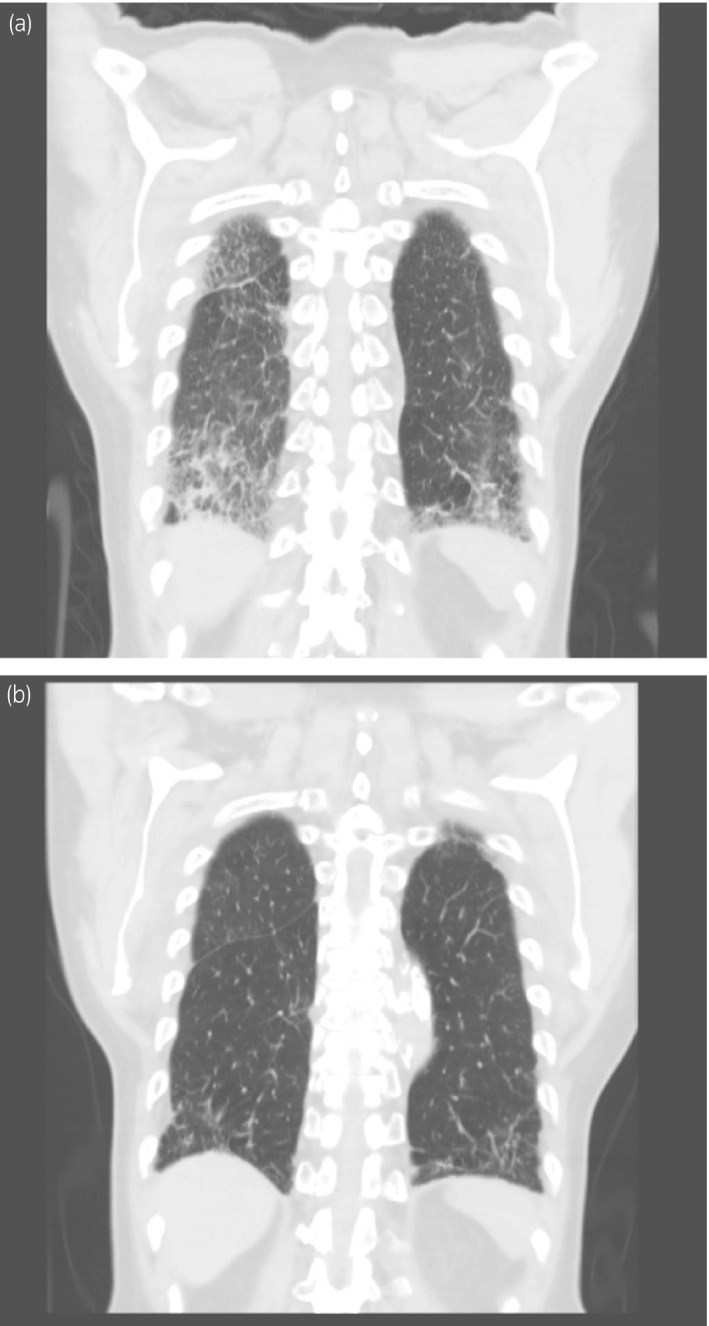
Appearance of chest CT scans of the ILD induced by apalutamide therapy. The apalutamide‐induced ILD (a) was successfully recovered by the steroid therapy (b)

Thirty‐five days after apalutamide therapy was initiated, the patient presented with dyspnea. His oxygen saturation (SpO_2_) in room air was found to be 87% by pulse oximetry. His serum KL‐6 level was 485 U/mL (normal range, <500 U/mL). Serum levels of procalcitonin, (1,3)‐beta‐D‐glucan, and various markers of collagen disease were within the normal reference range. The 2019 novel Coronavirus (2019‐nCoV) real‐time reverse transcriptase diagnostic panel also revealed a negative test result. Chest CT showed diffuse bilateral interstitial infiltrates and ground‐glass opacities in the upper and lower lobes of the lungs (Fig. 1a). Based on the absence of any suspect drug other than apalutamide and the characteristic findings, apalutamide was determined to be the cause of the ILD. Apalutamide was discontinued immediately, and intravenous steroid therapy with methylprednisolone (0.5 mg/kg/day) was administered for 2 weeks. Following this, the patient’s dyspnea improved, SpO_2_ level increased to 97% while breathing room air, and the ground‐glass opacity disappeared. The dosage of methylprednisolone was gradually tapered each month thereafter. Currently, the patient is undergoing follow‐up observation with LH‐RH agonist alone, and no increase in PSA level (PSA: 1.5 ng/mL) has been observed and bilateral interstitial infiltrates and ground‐glass opacities in the upper and lower lung lobes have improved in CT (Fig. 1b).

## Discussion

The exact incidence of drug‐induced ILD is not known. The number of reports of drug‐induced ILD has been on the rise in Japan in recent years. In general, Japanese people are severely susceptible to drug‐induced ILD, more than Europeans, Americans, and other Asians; thus, it has been suggested that genetic factors may be involved in the development of this disease.^8^ However, ILD due to prostate cancer therapy is rare, although it has been seen to be associated sporadically with first‐generation antiandrogen agents (bicalutamide and flutamide). ILD caused by second‐generation antiandrogens has been reported only in two cases, one each with abiraterone and apalutamide; therefore, it is extremely rare.^6^, ^7^ This is the second case report of ILD caused by apalutamide.

Regarding the clinical trials of apalutamide, the SPARTAN study showed that 2 of 803 patients (0.2%) in the apalutamide group had symptoms of dyspnea, with both being classified as grade 2 or lowery (according to the National Cancer Institute Common Terminology Criteria for Adverse Events, version 4.0).^9^ The TITAN study results demonstrated that none of the 524 patients in the apalutamide group developed respiratory symptoms.^10^

In general, drug‐induced ILD tends to occur between 2–3 weeks and 2–3 months after drug administration.^8^ For apalutamide, drug‐induced ILD was seen within 3 months in this case and in previously published literature.^11^, ^12^

When diagnosing suspected cases of drug‐induced lung injury, assessing subjective symptoms, along with SpO_2_, the timing of drug initiation or change, drug dosage, and treatment duration is beneficial. If respiratory symptoms and decreased SpO_2_ are observed, chest X‐ray and CT scans should be performed. However, since imaging studies do not relay specific findings, they cannot be used to confirm a diagnosis. A comparison of current imaging findings to those taken before drug initiation, as well as testing of serum markers and respiratory function, should be performed to identify exacerbations of pre‐existing pulmonary disease or infection. Serum levels of markers, such as KL‐6, surfactant protein‐A (SP‐A), and surfactant protein‐D (SP‐D), are important assessors of drug‐induced lung injury. At the onset of disease, if the serum KL‐6 level is within the normal range, follow‐up testing is recommended to assess the baseline value, as any variation is indicative of drug‐induced lung injury.^8^ In the present case, the patient’s KL‐6 level was within the normal range at the onset of ILD; however, the KL‐6 level decreased as symptoms improved after steroid therapy.

The DLST is a widely used method in Japan to identify drugs that cause side effects. The rate of DLST positivity in cases of diagnosed drug‐induced lung injury is low (55–70%)^8^, ^11^; therefore, it is not appropriate to solely rely on DLST results to rule out suspected causative drugs in lung injury cases. In this case, the DLST was not performed because of its low sensitivity and the steroid therapy had been started.

Risk factors for drug‐induced ILD are widely known, including increased age of 60 years or older, history of smoking, pre‐exiting lung disease, prior chemotherapy or thoracic radiotherapy, renal dysfunction, and diabetes mellitus.^11^ No references regarding risk factors for ILD with antiandrogenic drugs could be identified. In this patient, the common factors were age 60 years or older and smoking history.

In the previous report and our report of ILD with apalutamide, the patients were both Japanese.^7^ Although apalutamide has only been on the market for a short time, all reports of apalutamide‐induced ILD have been seen in Japanese patients. As has been reported in the previous cases of gefitinib‐induced ILD in Japanese patients, racial differences in the incidence of apalutamide induced ILD are likely to become clearer as more cases are reported.^11^

## Author Contributions

Fumiaki Kirishima: Conceptualization; Data curation; Formal analysis; Funding acquisition; Resources; Software; Supervision; Validation; Visualization; Writing – original draft. Yoshinori Shigematsu: Formal analysis; Project administration; Resources. Kanao Kobayashi: Investigation; Methodology; Project administration; Writing – review & editing.

## Conflict of interest

The authors declare no conflict of interest.

## Compliance with ethical standards

All procedures performed in this study involving human participants were in accordance with the ethical standards of the institutional and/or national research committee and with the 1964 Helsinki declaration and its later amendments or comparable ethical standards.

## Approval of the research protocol by an Institutional Reviewer Board

The protocol for this research project has been approved by the individual orally. And this is documented in the clinical record.

## Informed consent

Not applicable.

## Registry and Registration No. of the study/trial

Not applicable.
